# Continuous Gum Work

**Published:** 1856-04

**Authors:** C. S. Putnam

**Affiliations:** 35 Bond-st.


### ARTICLE XXI.

### *Continuous Gum Work.*

To the Editor Dental Recorder:

The frequent applications made to me for a minute statement of a system for using the body and gum materials, I prepare with "*positive* and *certain* success in *every case*," induces me to place the following before your readers:

In the preparation and combination of the ingredients composing the body, I find it necessary that all should be kept dry until put up (for sale,) and although every particle is sifted through a No. 11 bolting cloth, and is made much finer than many suppose necessary, yet it is unfit for use unless ground in water, in small quantities, *twenty-five to thirty minutes.*

The reason for this is simply as follows: two important ingredients composing it are entirely different from the remaining dozen, and each fusing at different degrees of heat from all the rest, their virtues or properties are lost before the whole is vitrified by fusion upon the piece of work, unless ground in water as before stated.

The grinding in water appears to intermix and combine the whole when baked into one, and an entirely different compound from any of the separate ingredients, or even the final combination, (if not thus ground,) and gives it the solidity and firmness upon the plate and around the teeth, so desirable for good work.

The body should be worked thoroughly between the teeth, and compressed until hard and close as pipe clay. The piece should be moved slowly into the muffle and remain at least *one-half hour*, at a *steady red heat*, after which the heat may be raised to a few degrees above the melting point of pure gold, and the set withdrawn to cool in a close extra muffle.

If baked in a quick or high heat, the surface will glaze, while the inner portion remains but slightly vitrified. If a second coat of body is required, it should be treated as the first.

If possible, nothing, not even the hand, should be brought in contact with the body after it is baked, before applying the gum. A particle of grease or dirt upon it may prevent a perfect reunion.

*The gum* is always to be used as put up, and should be laid on even, and quite moist, aud always left so as to require no carving or trimming except what may be necessary around the necks of the teeth.

If a layer is put over the first, no matter how thin, it is liable to flake up in baking. The same would occur if the gum is pressed or moved after it is first put on. Clock spring spatulas are found most useful in applying it. The piece should be heated up slowly and with care, but fused at a quick heat, *not exceeding four to five minutes.*

The *enamel* for "etched teeth," is to be moistened to the consistence of cream, and applied evenly upon the tooth (thick or thin as required) with a sable hair pencil.

It may be put on at the second baking of the body, or with the gum; its properties being almost entirely equivalent to the latter, minus the color.

Respectfully, &c.,

C. S. Putnam, 35 Bond-st.

[*Dent. Rec.*

